# Stopping Tuberculosis at the Gate: The Role of *M. tuberculosis* Adhesins in Infection and Intervention

**DOI:** 10.3390/vaccines13070676

**Published:** 2025-06-24

**Authors:** Haoyan Yang, Yinuo Ma, Xinkui Lei, Siyu Chai, Sigen Zhang, Guimin Su, Songping Li, Lin Du

**Affiliations:** 1 Research and Development Centre, Beijing Zhifei Lvzhu Biopharmaceutical Co., Ltd., Beijing 100176, China; yanghaoyan@zhifeishengwu.com (H.Y.); 17713140467@163.com (Y.M.); leixinkui@zhifeishengwu.com (X.L.); chaisiyu@zhifeishengwu.com (S.C.); zhangsigen@zhifeishengwu.com (S.Z.); suguimin@zhifeishengwu.com (G.S.); 2 Beijing Bacterial Vaccine Engineering Research Centre, Beijing 100176, China; 3 School of Life Science and Biopharmaceutics, Shenyang Pharmaceutical University, Shenyang 110016, China

**Keywords:** tuberculosis, *M. tuberculosis*, adhesin, computational prediction, infection prevention, drug resistance

## Abstract

The global burden of tuberculosis (TB), exacerbated by the rise of drug-resistant *Mycobacterium tuberculosis* (*M. tuberculosis*), underscores the need for alternative intervention strategies. One promising approach is to block the infection at its earliest stage—bacterial adhesion to host cells—thereby preventing colonization and transmission without exerting selective pressure. Adhesins, surface-exposed molecules mediating this critical interaction, have therefore emerged as attractive targets for early prevention. This review outlines the infection process driven by bacterial adhesion and describes the architecture of the *M. tuberculosis* outer envelope, emphasizing components that contribute to host interaction. We comprehensively summarize both non-protein and protein adhesins, detailing their host receptors, biological roles, and experimental evidence. Recent progress in the computational prediction of adhesins, particularly neural network-based tools like SPAAN, is also discussed, highlighting its potential to accelerate adhesin discovery. Additionally, we present a detailed, generalized workflow for predicting *M. tuberculosis* adhesins, which synthesizes current approaches and provides a comprehensive framework for future studies. Targeting bacterial adhesion presents a therapeutic strategy that interferes with the early stages of infection while minimizing the risk of developing drug resistance. Consequently, anti-adhesion strategies may serve as valuable complements to conventional therapies and support the development of next-generation TB vaccines and treatments.

## 1. Introduction

TB remains one of the most significant infectious diseases worldwide. In 2023, TB was expected to once again become the leading cause of death from a single infectious agent, after being temporarily overtaken by COVID-19. Over 10 million people contract TB annually, and the rise of drug-resistant *Mycobacterium tuberculosis* strains complicates treatment efforts. To eliminate TB by 2030, the United Nations (UN) and the World Health Organization (WHO) have endorsed a global strategy that requires improved screening, treatment, and novel therapeutic approaches [1,2].

One promising strategy focuses on the initial stages of infection, particularly the adhesion of *M. tuberculosis* to host cells. Adhesion plays a key role in pathogenesis. It allows bacteria to colonize host tissues, form biofilms, and resist stress [3,4,5,6]. *M. tuberculosis* adhesins bind to specific host receptors. This promotes bacterial attachment and colonization, both of which are critical for infection persistence [7,8]. The surface properties of the bacterium, such as surface free energy, charge, and hydrophobicity, also influence these interactions [9].

Targeting bacterial adhesion offers a novel approach for TB treatment. Anti-adhesion therapies can prevent bacterial attachment, reducing colonization, enhancing treatment effectiveness, and minimizing drug resistance [10,11]. For instance, in other pathogens such as *Helicobacter pylori* and uropathogenic *Escherichia coli* (UPEC), anti-adhesion agents like bovine lactoferrin (BLF) and FimH inhibitors have shown promise in improving infection outcomes [12,13,14,15,16]. These findings suggest that similar strategies could be effective in combating *M. tuberculosis*. Supporting this, serum from tuberculosis patients containing IgM antibodies against the adhesin HBHA has been shown to neutralize the bacterium’s entry into epithelial cells, implying that adhesin-specific immunity may offer protection against extrapulmonary dissemination. This evidence highlights the therapeutic potential of targeting adhesins, even in intracellular pathogens such as *M. tuberculosis* [17].

Despite advances in understanding *M. tuberculosis* adhesins, significant gaps remain. This review focuses on the mechanisms by which *M. tuberculosis* adheres to host cells, the role of important adhesins in infection, and their protective effects. While the prediction of adhesins in *M. tuberculosis* is still an emerging area of research, we will briefly outline the principles behind current prediction methods and highlight relevant findings from existing studies. By consolidating this knowledge, we aim to support the development of targeted therapies that could improve the treatment and prevention of TB.

## 2. Spatiotemporal Dynamics of Mycobacterial Adhesion During Infection

The adhesion of *M. tuberculosis* plays a critical role throughout its lifecycle, beginning with the inhalation of aerosol droplets containing the bacteria, which are expelled through the cough of an infected individual. Due to their small size, these droplets bypass the upper respiratory tract’s microbial flora and immune defenses, allowing *M. tuberculosis* to reach the alveolar spaces of the lungs [18,19]. Once in the alveoli, the bacteria are engulfed by alveolar macrophages (AM), initiating a complex immune response that leads to the formation of granulomas, structures that contain the bacteria in a latent state [20].

The initial adhesion of *M. tuberculosis* is mediated by surface-exposed lipids and glycoconjugates that interact with specific receptors on host cell surfaces, primarily on macrophages and alveolar epithelial cells (AECs). This process facilitates the bacteria’s attachment and entry into the host cells [21,22]. However, more recent studies have shown that *M. tuberculosis* may also utilize specialized adhesins for tissue dissemination. For example, *Mycobacterium bovis Bacillus Calmette-Guérin* (*M. bovis* BCG), as well as *M. tuberculosis*, uses heparin-binding hemagglutinin adhesin (HBHA), which binds to heparan sulfate-containing receptors on epithelial cells, aiding in the bacteria’s spread beyond the lungs [23]. This suggests that adhesins may help *M. tuberculosis* cross the alveolar epithelium, a process that may also occur via the transmigration of infected macrophages [24].

After the bacteria adhere to macrophages and AECs, the immune response can lead to the formation of granulomas, which contain the bacteria in a latent phase. In immunocompromised individuals, or children, the bacteria can undergo intense intracellular multiplication, which may lead to the breakdown of granulomas or the reactivation of latent infections. This can result in the dissemination of bacteria to distant sites, including the nervous system, bones, genitourinary system, and skin [25].

**Figure 1:**
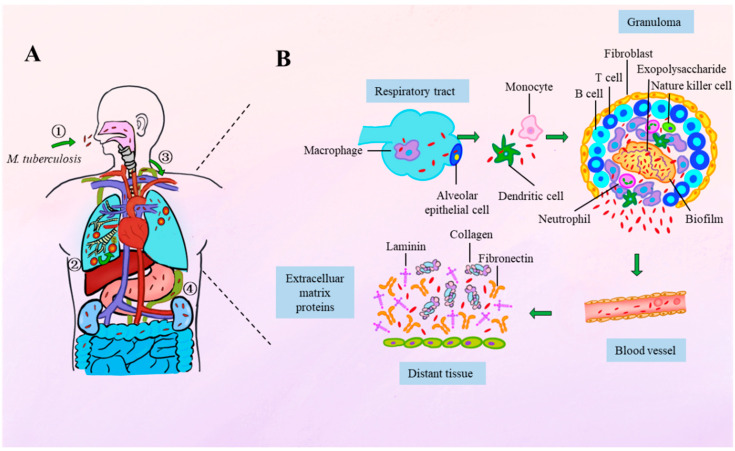
Spatiotemporal dynamics of *M. tuberculosis* infection progression and bacterial adhesion. (**A**) *M. tuberculosis* infection progression. (1) Transmission: TB is primarily contracted through inhalation of air containing *M. tuberculosis*. (2) Initial pulmonary infection: Inhaled bacilli reach the alveoli and are phagocytosed by alveolar macrophages, initiating a local immune response and leading to the formation of granulomas. These granulomas serve as a containment strategy but may also harbor persistent bacteria. (3) Lymphatic spread: Once the bacteria exit the lungs, they enter the lymphatic system and are likely to reach the circulatory system via the thoracic duct, which drains into the subclavian vein. (4) Extrapulmonary dissemination: Hematogenous spread allows the bacteria to colonize distant organs, leading to extrapulmonary tuberculosis. (**B**) Adhesion mechanisms throughout infection. Pulmonary infections initiate with adhesion to alveolar macrophages or epithelial cells. Following the migration across the alveolar epithelium, bacilli adhere to monocytes or dendritic cells. The immune response to the infection can sequester the bacteria within granulomas, where recent evidence has identified the presence of *M. tuberculosis* biofilms. Upon the breakdown of granulomas, the bacteria can attach to extracellular matrix (ECM) components. During the hematogenous spread, tubercle bacilli can bind plasma fibronectin, ultimately adhering to ECM proteins to facilitate invasion into new tissues.

During hematogenous spread, *M. tuberculosis* likely adheres to extracellular matrix (ECM) components to overcome mechanical shear forces and promote colonization in new tissues. For example, during hematogenous dissemination, the bacteria may bind plasma fibronectin and subsequently ECM proteins to invade new tissues (Figure 1) [26].

Thus, the adhesins of *M. tuberculosis* play an essential role not only in the initial stages of infection in the lungs but also in the subsequent spread and persistence of infection in distant organs. These adhesins enable the bacteria to cross epithelial barriers, invade new tissues, and evade immune clearance, making them key determinants of pathogenicity and transmission. Furthermore, adhesion is also a prerequisite for the formation of biofilms, which have been observed within granulomatous lesions. In this context, adhesins contribute to the establishment and stability of biofilm-like structures, which provide a protective microenvironment that enhances bacterial survival, limits antibiotic penetration, and promotes persistence during chronic infection [5]. This link between adhesion and biofilm formation underscores the broader role of adhesins in *M. tuberculosis* pathogenesis and highlights their potential as targets for disrupting both colonization and long-term persistence.

## 3. The *M. tuberculosis* Capsule: A Reservoir for Adhesins

**Figure 2:**
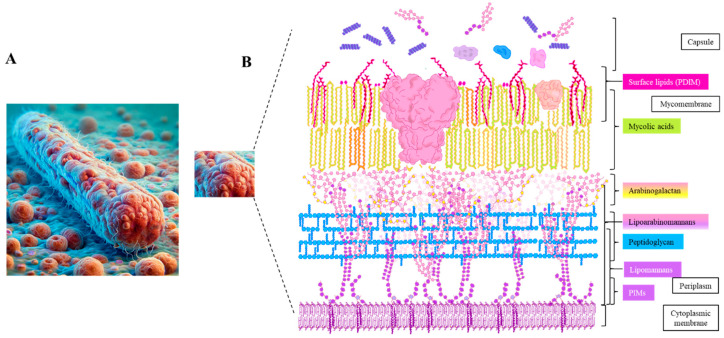
Schematic model of the *M. tuberculosis* cell envelope. (**A**) General illustration of the *M. tuberculosis* bacterial cell. (**B**) Detailed view of the multilayered architecture of the mycobacterial cell envelope. Phosphoinositol mannosides (PIMs), shown in light and dark purple, are short glycolipids consisting of an inositol core and mannose residues. Lipomannans (LMs) are elongated derivatives of PIMs, while lipoarabinomannans (LAMs) further incorporate branched arabinose chains (depicted in pink). Peptidoglycan is represented in blue, arabinogalactan in yellow (galactan) and pink (arabinan). Trehalose molecules, illustrated as paired dark pink hexagons, are conjugated with mycolic acids to form trehalose monomycolate (TMM) and trehalose dimycolate (TDM), key components of the outer lipid barrier. Mycolic acids are indicated in orange and light green, representing structurally distinct forms with different cyclopropane ring modifications or functional groups. Phthiocerol dimycocerosate (PDIM), a virulence-associated lipid, is marked in dark red. The blue-purple spiral represents α-glucan, while the remaining protein-like structures correspond to cell envelope proteins. Other classes of lipids and membrane components are not shown in this model for simplicity.

The bacterial cell envelope provides mechanical rigidity, facilitates solute and protein transport, and protects against environmental stresses. It consists of several layers, including the cytoplasmic membrane, cell wall, surface lipids, and capsule (Figure 2). The capsule, which is the outermost structure, contains key components such as lipids, glycoconjugates, and proteins. Many of these components function as adhesins, playing a crucial role in the bacterium’s ability to adhere to host receptors and establish infection [27].

### 3.1. Cytoplasmic Membrane

The mycobacterial cytoplasmic membranes contain glycerophospholipids, along with phosphatidylinositol mannosides (PIMs), lipomannans (LMs), and lipoarabinomannans (LAMs), which are derivatives of PIMs. While primarily contributing to cell envelope permeability and antibiotic resistance, PIMs, LMs, and LAMs also play a role in adhesion [28]. These molecules interact with host cells and influence immune responses, potentially affecting bacterial attachment and immune evasion [29].

### 3.2. Peptidoglycan (PG)

PG is a fundamental component of the mycobacterial cell wall, providing structural integrity and resistance to osmotic pressure. It consists of linear chains of alternating N-acetylmuramic acid and N-acetylglucosamine (GlcNAc), which are crosslinked by peptides to form a layered, honeycomb-like structure [30]. Beyond its structural role, PG is actively involved in host–pathogen interactions. Mycobacteria produce a unique form of peptidoglycan-derived muramyl dipeptide (MDP), known as N-glycolyl MDP, which enhances immune recognition by activating the pattern recognition receptor NOD2 [31]. Additionally, PG levels remain stable across different growth phases, suggesting that it may contribute to bacterial persistence within the host [32]. By engaging immune receptors and influencing granuloma formation, PG may play a role in bacterial adhesion and long-term survival during infection.

### 3.3. Arabinogalactan (AG)

The middle layer of the mycobacterial cell wall core consists of arabinogalactan sugars, which are linked to peptidoglycan via a rhamnose-GlcNAc disaccharide [32]. Beyond its structural role, AG has been identified as a virulence factor in *M. tuberculosis*, contributing to infection severity. Recent findings indicate that AG interacts with host receptors, including Galectin-9, a β-galactoside-binding protein, which activates the TAK1-ERK-MMP signaling pathway and leads to pathological changes in the lung [33,34]. This interaction suggests that AG may also play a role in bacterial adhesion, potentially influencing host–pathogen interactions during infection.

### 3.4. Mycolic Acids (MAs), Trehalose Monomycolate (TMM), and Trehalose 6,6′-Dimycolate (TDM)

Mycolic acids (MAs) are long-chain fatty acids (C60-C90) that form a major component of the mycobacterial cell envelope. They serve as the structural backbone of the mycomembrane by covalently linking to the arabinogalactan–peptidoglycan (AG-PG) matrix. MAs also exist on the bacterial surface in free or glycosylated forms, such as TMM and TDM [35]. Beyond their structural role, MAs and their derivatives may contribute to mycobacterial adhesion. As key components of the outermost layer of the bacterial envelope, they provide a direct interface with the host. While the precise mechanisms remain unclear, MAs have been implicated in interactions with host immune receptors such as Mincle and TREM2, which may modulate immune recognition and bacterial attachment [36]. Given their surface localization and involvement in host–pathogen interactions, MAs and their derivatives could play a role in facilitating bacterial colonization and dissemination during infection.

### 3.5. Phthiocerol Dimycocerosate (PDIM)

PDIM is a key surface lipid of *M. tuberculosis*, playing an essential role in both immune evasion and bacterial adhesion. PDIM helps the bacteria evade the innate immune system by masking pathogen-associated molecular patterns (PAMPs) on the cell wall, thus preventing the activation of immune responses. This immune evasion enables the recruitment of permissive monocytes, which are more favorable for bacterial survival, rather than microbicidal macrophages [37]. In addition to immune modulation, PDIM may also facilitate adhesion. Its methyl-branched fatty acids increase lipid mobility, potentially enhancing bacterial attachment to host cells such as epithelial cells and macrophages. While PDIM primarily functions to hide bacterial PAMPs, its role in promoting initial bacterial attachment suggests a dual function in immune evasion and colonization [37,38].

## 4. *M. tuberculosis* Adhesins

Adhesins play a crucial role in *M. tuberculosis* attachment to and entry into host cells. They can be broadly classified into non-protein and protein adhesins, both of which interact with host receptors to mediate adhesion. *M. tuberculosis* adhesion is a multifactorial process that involves the simultaneous action of multiple adhesins, rather than a single dominant factor. Major surface adhesins of *M. tuberculosis* and their host receptors are listed in Table 1. The following sections provide a detailed overview of these adhesins and their corresponding host receptors.

### 4.1. Non-Protein Adhesins in the Mycobacterial Cell Wall and Their Host Interactions

#### 4.1.1. Mannose-Capped Lipoarabinomannan (ManLAM) and PIMs

ManLAM and PIMs, two key mannose-capped components of the *M. tuberculosis* cell wall, act as adhesins by binding to the dendritic cell-specific ICAM-3-grabbing non-integrin (DC-SIGN) and Dectin-2 receptors on immature dendritic cells and macrophages [39,41,42]. This interaction has been linked to both immune activation and immune evasion. On the one hand, the binding of manLAM to Dectin-2 on dendritic cells promotes the activation of T cell responses and enhances immunity, as seen in certain experimental models [42]. On the other hand, this interaction can also impair dendritic cell maturation and inhibit the immune response, contributing to the evasion of protective immunity against tuberculosis. Additionally, manLAM and higher-order PIMs prevent the fusion of phagosomes with lysosomes in macrophages, helping the bacteria avoid degradation [81]. However, studies on mycobacterial strains lacking manLAM or PIMs suggest that the manLAM/PIM-DC-SIGN interaction is not essential for cytokine release in vitro or for protective immunity in vivo, indicating that these adhesins might be more important for bacterial survival and immune modulation rather than directly triggering immune responses [41,82].

#### 4.1.2. MAs

MAs, which are α-alkyl-β-hydroxy fatty acids, are the major lipid components of the *M. tuberculosis* cell wall. These hydrophobic molecules contribute significantly to the bacterial resistance to drugs and oxidative stress by forming a protective barrier. In addition to their structural role, MAs influence host–pathogen interactions. Experimental studies show that MAs can directly interact with macrophages, affecting both cell survival and immune response [83]. Specifically, MAs at lower concentrations promote macrophage survival, while higher concentrations induce apoptosis by triggering the release of pro-apoptotic cytokines [83]. This suggests that MAs not only modulate immune responses but also interact with macrophages to influence their behavior.

Furthermore, MAs interact with immune molecules such as galectin-3 and Clec12A [45,46]. Galectin-3, a lectin involved in immune recognition, specifically binds to the mycolic acid components of the *M. tuberculosis* cell wall, which may facilitate bacterial adhesion and immune evasion [45]. Mycobacterial MAs also bind to the inhibitory C-type lectin receptor Clec12A, found on immune cells. This interaction suppresses immune responses, as evidenced by enhanced immune reactions in Clec12A-deficient mice after infection, and increased susceptibility in human Clec12A transgenic mice [46]. These findings underscore the diverse role of MAs in modulating the host immune response and suggest that targeting these interactions could offer new therapeutic approaches for combating mycobacterial infections.

#### 4.1.3. TDM

TDM, a key glycolipid in the *M. tuberculosis* cell wall, is an important adhesin in infection. It is recognized by several pattern recognition receptors (PRRs), including macrophage-inducible C-type lectin (Mincle), the macrophage receptor with a collagenous structure (MARCO), and Dectin-3. Mincle, a C-type lectin receptor, is essential for TDM-induced macrophage activation, promoting the release of pro-inflammatory cytokines and nitric oxide. In Mincle-deficient macrophages, these immune responses are completely suppressed, highlighting Mincle’s crucial role in TDM recognition. TDM also induces granuloma formation in vivo, a hallmark of mycobacterial infection, which is absent in mincle-deficient mice [47,48].

Dectin-3 also recognizes TDM and promotes Mincle expression, amplifying the immune response. In Dectin-3-deficient macrophages, this activation is significantly impaired, emphasizing Dectin-3’s importance in the TDM-induced immune response [49]. Additionally, MARCO, a class A scavenger receptor, binds TDM and activates the TLR2 signaling pathway, enhancing cytokine production. MARCO-deficient macrophages show a reduced immune response to TDM [50,51].

Together, these findings demonstrate that TDM, through its interactions with multiple receptors, plays a vital role in both the bacterial adhesion and modulation of the host immune system. These interactions not only facilitate *M. tuberculosis* colonization but also shape the host immune response, highlighting TDM as a key player in tuberculosis pathogenesis.

#### 4.1.4. α-Glucan

*M. tuberculosis* α-glucan functions as an adhesin and a novel ligand for the C-type lectin receptor DC-SIGN, with binding mediated by internal glucosyl residues. This interaction induces IL-10 production in LPS-activated monocyte-derived dendritic cells (DCs), contributing to immune suppression [52]. Additionally, α-glucan interferes with monocyte differentiation, preventing their maturation into fully competent DCs and promoting the development of CD1-negative dendritic cells (CD1⁻ DCs). By blocking class I CD1 molecule expression, α-glucan disrupts CD1-mediated antigen presentation, which is crucial for the activation of CD1-restricted lipid-specific T cells [53]. Since *M. tuberculosis* has a high content of antigenic lipids, this immune evasion mechanism may impair the host’s ability to mount an effective cell-mediated immune response. Notably, CD1-restricted T cell responses are significantly reduced in patients with active pulmonary tuberculosis, further highlighting the role of α-glucan-DC-SIGN interaction in *M. tuberculosis* pathogenesis [84].

### 4.2. Protein Adhesins in the Mycobacterial Cell Wall and Their Host Interactions

#### 4.2.1. Early Secretory Antigenic Target 6 kDa (ESAT-6) and Culture Filtrate Protein 10 kDa (CFP-10)

Although ESAT-6 and CFP-10 are classically characterized as secreted virulence factors, increasing evidence supports their additional role as non-canonical adhesins that mediate direct interaction with host cells [55,75]. ESAT-6 and CFP-10 form a stable 1:1 complex and are coordinately regulated during *M. tuberculosis* infection [54]. CFP-10 likely functions as a chaperone, stabilizing ESAT-6, which is implicated in disrupting phagosomal membrane integrity through an incompletely understood mechanism [85]. Beyond their roles in membrane disruption, ESAT-6 and CFP-10 also act as adhesins, facilitating bacterial invasion and dissemination within the host [86].

During infection, ESAT-6 is recognized as a PAMP that stimulates Toll-like receptors (TLRs), triggering innate immune responses [87]. In pneumocytes, surface-expressed ESAT-6 mediates bacterial adhesion to the basolateral laminin-expressing surface, contributing to host cell damage and basement membrane degradation. This interaction promotes bacterial dissemination through the alveolar wall [55]. Renshaw et al. demonstrated that the ESAT-6-CFP-10 complex directly mediates macrophage binding, enhancing bacterial uptake and survival [54]. Furthermore, studies in embryonic zebrafish suggest that ESAT-6 enhances the trafficking of infected macrophages within granulomas, facilitating early bacterial dissemination [86].

In vitro studies using an alveolar barrier model, composed of human alveolar epithelial and endothelial cells cultured on opposite sides of a basement membrane, revealed that strains lacking ESAT-6 exhibit reduced migration across this barrier [88]. These findings collectively support a model in which ESAT-6 and CFP-10 contribute to adhesion-mediated steps of infection, consistent with their inclusion in the current review as functionally relevant adhesins, despite their classification as secreted proteins.

#### 4.2.2. Antigen 85 (Ag85) Complex

The Ag85 complex in *M. tuberculosis* comprises three paralogous proteins—Ag85A, Ag85B, and Ag85C—which are secreted in large amounts and elicit robust humoral and cell-mediated immune responses [89]. Interestingly, despite their high secretion levels, a significant portion of these proteins remains associated with the bacterial surface, suggesting a potential role in host–pathogen interactions [90]. All three Ag85 proteins have been shown to bind fibronectin (Fn), a key extracellular matrix protein, highlighting their importance in mediating bacterial adhesion—a critical step in host invasion [56].

The functional relevance of the Ag85-Fn interaction has been demonstrated through the siRNA-mediated depletion of Fn, which significantly reduces the binding of purified Ag85B to human epithelial cells [91]. This finding is further supported by the identification of Ag85-Fn complexes in clinical samples from tuberculosis patients, underscoring the biological significance of this interaction in infection [92]. Notably, the specific amino acid residues that are critical for Fn binding have been mapped to the sequence ^98^FEWYYQSGLSV^108^ in Ag85B from *Mycobacterium kansasii.* This sequence appears to be unique among known prokaryotic and eukaryotic Fn-binding proteins, suggesting a distinct mechanism of interaction [93]. These findings highlight Ag85 as a key adhesin facilitating *M. tuberculosis* attachment to host tissues, contributing to its pathogenicity.

#### 4.2.3. HBHA

*M. tuberculosis* adheres to, invades, and proliferates in both professional phagocytes and epithelial cells. Among its adhesins, HBHA plays a crucial role in mediating adherence to epithelial cells and facilitating extrapulmonary dissemination [58]. HBHA specifically binds to sulfated glycoconjugates on epithelial cells, and the disruption of its synthesis significantly impairs bacterial dissemination from the lungs to other organs, such as the spleen [86].

Experimental studies have demonstrated the mechanistic role of HBHA in receptor-mediated endocytosis. Franco D. Menozzi and colleagues showed that HBHA, when conjugated to colloidal gold particles, rapidly attaches to the membranes of human laryngeal epithelial cells and type II pneumocytes. This process is dependent on the heparin-binding domain of HBHA, as gold particles coated with a truncated HBHA lacking this domain fail to bind. Furthermore, pretreatment of epithelial cells with heparinase III, an enzyme that selectively cleaves heparan sulfate chains, abolishes HBHA-mediated attachment [94]. These findings establish HBHA as a key adhesin that facilitates *M. tuberculosis* adherence to host epithelial cells through interactions with heparan sulfate-containing proteoglycans.

#### 4.2.4. *Mycobacterium Tuberculosis* Curli Pili (MTP)

MTP, encoded by the Rv3312A (mtp) gene, is a surface-associated adhesin that plays a critical role in host–pathogen interactions [95]. It facilitates the initial attachment of *M. tuberculosis* to host cells, a key step in establishing infection. MTP specifically binds to the extracellular matrix protein laminin and contributes to biofilm formation, which provides a reservoir for persistent bacterial cells that are resistant or tolerant to antibiotics [62].

Experimental studies have demonstrated the functional significance of MTP in adhesion and invasion. *M. tuberculosis* strains lacking *mtp* exhibit impaired MTP production in vitro and a significantly reduced laminin-binding capacity [63]. In macrophages, an *mtp* deletion mutant (*Δmtp*) displayed markedly reduced adhesion and invasion compared to the wild-type strain, while a complemented strain overexpressing *mtp* showed enhanced macrophage interaction [61]. Similarly, the loss of MTP significantly diminished the ability of *M. tuberculosis* to adhere to and invade A549 pulmonary epithelial cells. Complementation of the *mtp* mutant restored its adhesive and invasive properties to wild-type levels [60].

These findings establish MTP as a crucial adhesin that mediates *M. tuberculosis* interaction with host cells, promoting bacterial colonization and persistence.

#### 4.2.5. 19-kDa Antigen

The 19-kDa antigen of *M. tuberculosis* is a cell wall-associated lipoprotein that functions as an adhesin, facilitating bacterial interaction with host cells [96,97]. It is glycosylated and acylated, enabling its recognition by host receptors. Studies have demonstrated that the 19-kDa antigen preferentially binds to THP-1 macrophages, with binding inhibited by mannose receptor competitor sugars, calcium chelators, and monoclonal antibodies against the mannose receptor. This suggests that the mannose receptor mediates the adhesion of *M. tuberculosis* through this antigen. Additionally, screening assays using biotin-labeled culture filtrate proteins identified the 19-kDa antigen as a dominant adhesin, further supporting its role in host–pathogen interactions [65].

#### 4.2.6. Alanine and Proline-Rich Protein (Apa)

The Apa is a secreted adhesin of *M. tuberculosis* that plays a crucial role in host–pathogen interactions. Apa facilitates bacterial adhesion to host cells by binding to Fn, with key Fn-binding motifs identified within residues 269–280, particularly the conserved ^273^RWFV^276^ sequence. Blocking the Apa-Fn interaction significantly reduces bacterial attachment and invasion, highlighting its importance in mycobacterial pathogenesis [68]. Additionally, Apa interacts with pulmonary surfactant protein A (PSP-A), a C-type lectin involved in innate immune recognition. This binding is calcium- and mannose-dependent, as shown by the loss of interaction upon EDTA treatment or deglycosylation. These findings suggest that Apa functions as a multifunctional adhesin, mediating bacterial attachment through both fibronectin and PSP-A interactions [69].

#### 4.2.7. Mycobacterial Mammalian Cell Entry Protein 1A (Mce1A)

Mce1A is a mycobacterial adhesin that facilitates bacterial attachment and invasion into host cells. Studies using *Escherichia coli* expressing Mce1A have demonstrated enhanced bacterial adhesion and intracellular survival in human monocytes and alveolar epithelial cells, accompanied by increased MCP-1 and IL-8 production [73,74]. In *Mycobacterium leprae*, Mce1A mediates invasion into human microvascular endothelial cells (HMVEC), which serve as reservoirs before bacterial dissemination to Schwann cells. Blocking specific regions of Mce1A with antibodies significantly reduces bacterial entry, highlighting its essential function in host–pathogen interactions [72].

#### 4.2.8. PE_PGRS33 (Rv1818c)

PE_PGRS33 is a surface antigen of *M. tuberculosis* that functions as an adhesin, facilitating bacterial entry into macrophages [77]. The PE domain of PE_PGRS33 is essential for its translocation across the mycobacterial cell wall, while the PGRS domain plays a key role in interacting with host receptors [77,78].

In macrophages, this interaction occurs through Toll-like receptor 2 (TLR2) in a calcium-dependent manner, as evidenced by the failure of macrophages from TLR2-deficient mice to internalize the bacteria [77,79,98]. Notably, the PGRS domain, particularly the region spanning amino acids 140–260, is critical for mediating bacterial entry [78]. Supporting this, antibodies against PE_PGRS33 significantly reduce bacterial uptake, while *M. tuberculosis* strains lacking Rv1818c exhibit impaired entry. Furthermore, supplementing these mutant strains with recombinant PE_PGRS33 restores their ability to invade macrophages. Immunization with native recombinant PE_PGRS33 has also been shown to enhance bacterial restriction in vivo, highlighting its potential role in host–pathogen interactions and immune modulation [78,79].

## 5. Advances in In Silico Prediction of *M. tuberculosis* Adhesins

Before the application of computational techniques in predicting *M. tuberculosis* adhesins, researchers primarily relied on experimental methods to identify putative adhesins. This approach led to the identification of several adhesins, including HBHA, and PE_PGRS proteins [79,99]. However, experimental verification is labor-intensive and costly, limiting its feasibility for the development of novel tuberculosis vaccines. To address this challenge, computational techniques, which have been widely used in biopharmaceutical research, were introduced into this field.

Traditional methods, such as homologous sequence alignment and structural analysis, offer a preliminary means to identify potential adhesins. However, the lack of universally conserved motifs among bacterial adhesins and the limited number of functionally characterized proteins restrict the broader applicability of these methods [100]. With the emergence of reverse vaccinology and the availability of the complete *M. tuberculosis* genome, computational prediction has become a critical tool for elucidating protein function [101,102,103]. One such method is the Software Program for the Prediction of Adhesins and Adhesin-like Proteins using Neural Networks (SPAAN), which predicts adhesins based on non-homologous sequence features and supports accelerated vaccine research [104].

**Figure 3:**
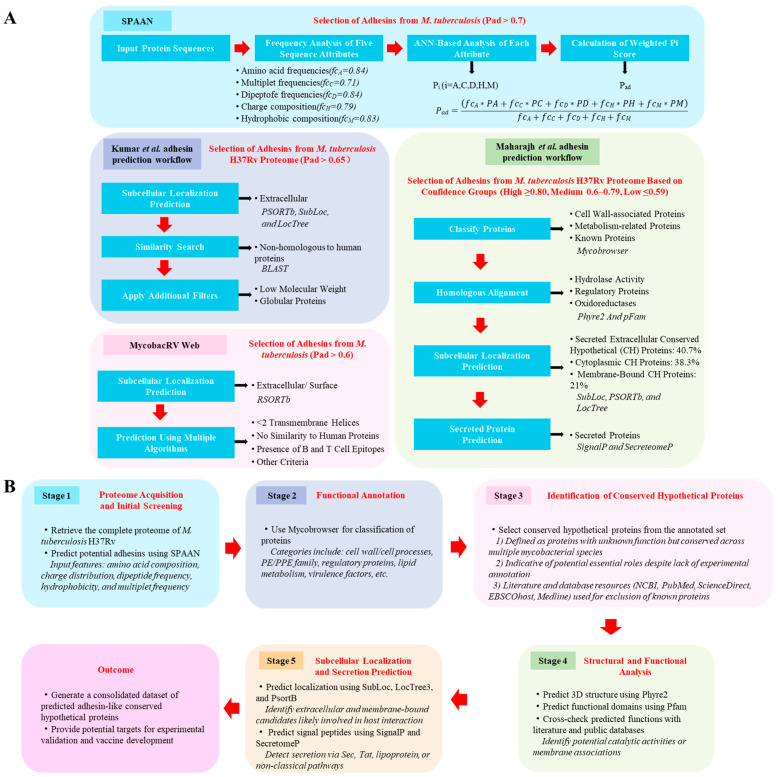
In silico prediction of *M. tuberculosis* adhesins. (**A**) Various in silico prediction methods for identifying *M. tuberculosis* adhesins described in the literature. (**B**) Bioinformatic pipeline for predicting adhesin-like proteins from *M. tuberculosis* using latest general prediction methods. This pipeline consists of five sequential stages. In Stage 1, the complete proteome of *M. tuberculosis* H37Rv is retrieved and screened using the SPAAN to predict potential adhesins based on amino acid composition and related features. In Stage 2, functionally annotated proteins are selected using Mycobrowser, focusing on categories relevant to host–pathogen interaction. Stage 3 refines the candidates by identifying conserved hypothetical proteins, defined as uncharacterized proteins conserved across mycobacterial species, while excluding known proteins using multiple databases. In Stage 4, structure prediction is performed using Phyre2, and functional domains are analyzed using Pfam, complemented by the literature-based functional assessment. Stage 5 involves subcellular localization and secretion prediction using tools such as SubLoc, PsortB, and SignalP, prioritizing extracellular and membrane-bound proteins. Each of the five steps is visually represented as a separate module in the diagram, with arrows indicating the flow of analysis.

The SPAAN evaluates 105 compositional features of proteins, grouped into amino acid frequencies, multiplet frequencies, dipeptide frequencies, charge composition, and hydrophobic composition. Each group is analyzed by an individual artificial neural network (ANN), and the combined outputs generate a final prediction score (P_ad_), representing the likelihood of a protein being an adhesin. The SPAAN model was optimized using iterative training with known adhesins and non-adhesins. A threshold of P_ad_ ≥ 0.51 was found to yield high predictive accuracy and specificity. Additionally, the SPAAN showed reliable performance when applied to viral adhesins, supporting its generalizability. For *M. tuberculosis*, a stricter P_ad_ threshold of >0.7 was suggested to reduce false-positive predictions, considering that HBHA, an experimentally confirmed adhesin, had a P_ad_ score of 0.6763 (Figure 3A).

Despite its strengths, the SPAAN has limitations. Its dependence on full-length amino acid sequences constrains its utility in cases with incomplete sequence data. Furthermore, the presence of false positives necessitates downstream validation using complementary computational or experimental approaches.

Since adhesins are typically located on the bacterial surface or secreted extracellularly to mediate interactions with host proteins, integrating subcellular localization predictions enhances adhesin identification [105,106]. Based on this rationale, Kumar et al. and the MycobacRV web server combined SPAAN outputs with localization tools such as PSORTb, SubLoc, and LocTree [71,107]. Recognizing that some experimentally validated adhesins had P_ad_ scores below 0.7, these studies used relaxed thresholds (P_ad_ ≥ 0.65 or 0.6) to capture a broader range of candidates (Figure 3A).

Kumar et al. applied the SPAAN to the entire *M. tuberculosis* H37Rv proteome and refined predictions through homology-based functional annotation, structural modeling, and evaluation of extracellular matrix-binding capacity [71]. The MycobacRV web server extended this pipeline to 23 *M. tuberculosis* strains and selected extracellular or cell wall-associated proteins using PSORTb v3.0 [107]. These predictions were further analyzed using immunoinformatics tools to identify antigenic adhesin epitopes as vaccine candidates.

These integrative approaches expanded the spectrum of predicted *M. tuberculosis* adhesins. For example, Kumar et al. identified 20 putative adhesins in H37Rv, and experimental validation confirmed the adhesin roles of Rv0309, Rv2599, and Rv3717 [71]. Similarly, MycobacRV identified 233 vaccine candidate sequences, including 37 novel adhesins in H37Rv, contributing to tuberculosis vaccine development [75].

While these studies focused on subsets of proteins, Maharajh et al. conducted a comprehensive evaluation of SPAAN-based predictions and proposed a systematic workflow for classifying uncharacterized H37Rv proteins [108]. Rather than using a fixed P_ad_ threshold, they stratified 167 predicted adhesins into high (≥0.80), medium (0.6–0.79), and low (≤0.59) confidence groups. Additional analyses, including homology modeling, localization, and secretion prediction, improved the robustness of the predictions. The use of a large dataset enhanced the credibility of SPAAN-based workflows (Figure 3A).

Among existing tools, the SPAAN program remains the foundation for adhesin prediction in *M. tuberculosis*, but its high false-positive rate necessitates further refinement. To improve prediction accuracy, Kumar et al. and the MycobacRV platform incorporated subcellular localization and domain-based information. Maharajh et al. proposed a more systematic SPAAN-based workflow, stratifying candidates into confidence levels and integrating additional bioinformatic evidence such as secretion signals and structural modeling.

Building upon these developments, we developed a stepwise prediction pipeline (Figure 3B) that offers a more detailed and user-friendly workflow. While largely based on the strategy proposed by Maharajh et al., our version expands the evaluation steps to include cross-referencing with public databases (e.g., PubMed, ScienceDirect) and literature mining, which may assist researchers in prioritizing adhesin candidates for experimental validation. This practical framework aims to facilitate more reproducible and transparent adhesin prediction in *M. tuberculosis* and potentially other pathogens.

## 6. Challenges and Controversies in Adhesin-Targeted Strategies

### 6.1. Functional Complexity and Contradictory Evidence

The infection process of *M. tuberculosis* is inherently complex and often paradoxical. Granuloma formation, while primarily intended to contain infection, also contributes to tissue damage and bacterial persistence [109]. Similarly, cytokines induced during infection can exhibit both protective and pathogenic roles depending on the immune context [110]. These examples reflect the broader challenge of interpreting immune responses to *M. tuberculosis*, where host-beneficial and pathogen-favoring mechanisms frequently coexist.

This complexity is also evident in the study of adhesins. Although adhesins are recognized as promising targets for TB intervention, their functions are not always straightforward. A well-characterized example is ManLAM, which interacts with receptors such as DC-SIGN and Dectin-2 on dendritic cells and macrophages. These interactions have been reported to either promote antigen presentation and T cell priming or inhibit dendritic cell maturation and facilitate immune evasion [42,81]. Several factors may contribute to these contradictions: the function of an adhesin may vary depending on the stage of infection; the same adhesin–receptor interaction may trigger distinct outcomes in different host cell types; and structural variations in the adhesin itself—such as differences in glycosylation or lipid composition—may influence its immunological effects.

A more balanced interpretation of these findings—acknowledging both the immunostimulatory and immunosuppressive outcomes of adhesin–host interactions—is essential for advancing our understanding of their roles in pathogenesis and for informing rational vaccine and therapeutic design. Future studies should therefore incorporate infection-stage-specific models, single-cell immune profiling, and detailed structure–function analyses to clarify the precise biological functions of adhesins under physiologically relevant conditions.

### 6.2. Translational and Practical Challenges

Despite the conceptual appeal of targeting *M. tuberculosis* adhesins as a prophylactic or therapeutic strategy, several translational and practical barriers remain. First, adhesins are not essential for bacterial viability, and their immunogenicity may not suffice to induce sterilizing immunity, particularly in individuals with latent or active tuberculosis. As such, anti-adhesin approaches are most suitable for pre-exposure prophylaxis, aiming to block bacterial colonization at the earliest stage of infection. By interfering with host–pathogen interactions rather than bacterial survival, these strategies exert minimal selective pressure, which could be advantageous in reducing the risk of drug resistance. However, their application in post-infection settings remains limited. In such cases, adhesin-targeted interventions may help restrict dissemination or reinfection, particularly when integrated into combination therapies that also target intracellular survival and persistence mechanisms.

Another major obstacle is the high degree of functional redundancy among *M. tuberculosis* adhesins. The bacterium expresses a large array of surface-exposed ligands—both proteinaceous and non-proteinaceous—many of which bind overlapping host targets or operate synergistically. Targeting a single adhesin may be insufficient due to compensatory mechanisms, making broad protection difficult to achieve with monovalent formulations. Additionally, the development of effective delivery platforms that ensure antigen stability, accessibility, and proper immune engagement remains a practical challenge.

### 6.3. Strategic Solutions and Future Directions

Addressing these challenges will require a combination of rational target selection and innovative technological approaches. One critical step is the identification of conserved, immunogenic, and surface-accessible adhesins that are expressed during key stages of infection. Spatiotemporal mapping of adhesin expression may help distinguish constitutively expressed adhesins from those that are only induced in specific niches, guiding more precise targeting strategies. The construction of cross-species adhesin databases could further facilitate the discovery of broadly relevant antigens, while CRISPR-based genome editing and high-throughput interaction screens may support the functional validation and ranking of candidate targets.

To overcome the limitations of individual adhesins, multivalent vaccines incorporating multiple key adhesins—either as fusion proteins, multi-epitope constructs, or mRNA-encoded cocktails—are under consideration. These could be further optimized by co-delivery with antigens expressed during later stages of infection, such as ESAT-6 or Ag85B, thereby enhancing coverage across the entire infection timeline. Such combined approaches may enable adhesin-based strategies to serve both prophylactic and adjunct therapeutic roles. Continued integration of computational prediction, structural modeling, and experimental validation will be essential for translating adhesin research into clinically viable interventions.

## 7. Conclusions and Future Perspectives

*M. tuberculosis* infection begins with a well-coordinated adhesion process. Multiple adhesins mediate attachment to host cells and extracellular matrix components. This early step enables the pathogen to evade clearance, establish granulomas, and eventually disseminate to distant tissues. This review outlines the complex architecture of the mycobacterial cell envelope. These structures facilitate host receptor interactions and contribute to immune modulation. We also highlighted the diverse types of *M. tuberculosis* adhesins—including proteins, glycolipids, and other non-protein components—and summarized their respective roles in host colonization. We also reviewed advances in computational adhesin prediction. SPAAN-based pipelines have enabled the large-scale identification of candidate adhesins in multiple *M. tuberculosis* strains.

Targeting bacterial adhesion offers a promising strategy for tuberculosis prevention and treatment, especially in light of rising drug resistance. Unlike bactericidal drugs, adhesion inhibitors function by preventing bacterial attachment rather than by killing the pathogen. This approach minimizes selective pressure and may help reduce the emergence of resistant strains. Since adhesion is not essential for bacterial survival, these strategies may preserve commensal microbiota. They may also reduce inflammation caused by bacterial cell lysis. Preclinical studies have demonstrated that antibodies or receptor mimetics targeting major adhesins—such as HBHA, Apa, and the 19-kDa protein—can block *M. tuberculosis* attachment and infection. However, due to the extensive repertoire of adhesins expressed by *M. tuberculosis*, effective therapeutic interventions may require a multi-target approach. Identifying and validating a core set of adhesins that contribute most significantly to virulence and host interactions will be critical for advancing this strategy.

Looking ahead, integrating computational prediction, structural biology, and experimental validation will be essential. This will help map the *M. tuberculosis* adhesome more completely. A deeper understanding of the spatial and temporal expression of adhesins during different stages of infection may reveal new opportunities for targeted intervention. Furthermore, the development of adhesin-based vaccines or adjunct therapies could complement existing tuberculosis treatment regimens, shorten therapy duration, and improve outcomes for drug-resistant TB cases. As the field moves forward, adhesins hold significant potential as both diagnostic markers and novel therapeutic targets in the global fight against tuberculosis.

## Figures and Tables

**Table 1:** Key mycobacterial adhesins and their host receptors.

No.	Adhesin	Receptor	Phenotype During Infection	Refs.
1	Phosphatidylinositol-mannosides (PIMs)	Dendritic cell-specific ICAM-3-grabbing non-integrin (DC-SIGN)	PIMs contribute to the interaction between mycobacteria and dendritic cells, although other unknown ligands may be more dominant in this process.	[39]
		Galectin-3	Infection of galectin-3-deficient mice with *M. tuberculosis* reveals a reduced capacity to clear late-stage, but not early-stage, infection.	[40]
2	Mannose-capped lipoarabinomannan (manLAM)	DC-SIGN	ManLAM’s mannose cap is thought to inhibit phagolysosome fusion and promote IL-10 production, but this effect is less important in interactions with living bacteria.	[41]
		Dectin-2	Dectin-2 deficiency exacerbates lung pathology during mycobacterial infection in mice.	[42,43,44]
3	Arabinogalactan (AG)	Galectin-9	Deletion of galectin-9 blocks AG-induced lung pathology, and the AG-galectin-9 axis exacerbates *M. tuberculosis* infection in mice.	[34]
4	Mycolic acid (MA)	Galectin-3	N.A.	[45]
		Clec12A	Clec12A-deficient mice show enhanced innate and T cell responses after infection, while human Clec12A transgenic mice are more susceptible to *M. tuberculosis* infection.	[46]
5	Trehalose 6,6′-dimycolate (TDM)	Mincle	In vivo administration of TDM triggers a significant increase in inflammatory cytokines in serum and induces characteristic lung inflammation, including granuloma formation.	[47,48]
		Dectin-3	TDM stimulation of Dectin-3 induces Mincle expression, potentially boosting the host’s innate immune response to Mycobacterium infection.	[49]
		Macrophage receptor with collagenous structure (MARCO)	MARCO-expressing macrophages secrete pro-inflammatory cytokines in response to TDM, while macrophages from MARCO(−/−) mice produce significantly lower cytokine levels upon infection with virulent *M. tuberculosis*.	[50,51]
6	α-glucan	DC-SIGN	Inhibits DC maturation and suppresses DC-mediated immune responses; additionally, induces IL-10 secretion.	[52,53]
7	Early secretory antigenic target 6 kDa (ESAT-6) *, culture filtrate protein 10 kDa (CFP-10) *	N.A.	The binding of the fluorescently labeled ESAT-6-CFP-10 complex to the surface of macrophages is directly mediated by the protein complex. Moreover, the flexible C-terminal arm of CFP-10 plays a crucial role in this interaction, forming an essential part of the binding site for the cell surface receptor.	[54]
8	ESAT-6 *	Laminin	ESAT-6 induces cytolysis in both type 1 and type 2 pneumocytes.	[55]
9	Antigen 85 (Ag85) complex	Fibronectin (Fn)	Fn depletion through siRNA significantly impaired the binding of purified Ag85B to human epithelial cells, highlighting the crucial role of the Ag85B-Fn interaction in epithelial adhesion.	[56]
	Ag85B	Plasminogen (Plg)	Ag85B binds to human Plg, and promotes its activation to plasmin (Plm), potentially enhancing the extracellular matrix degradation and tissue invasion of *M. tuberculosis*.	[57]
10	Heparin-Binding Hemagglutinin Adhesin (HBHA)	Heparan sulfate glycosaminoglycans	Facilitates bacillary adhesion to host cells and promotes epithelial transcytosis, enabling *M. tuberculosis* to establish systemic infection.	[58,59]
11	*Mycobacterium tuberculosis* curli pili (MTP)	N.A.	An MTP-deficient strain of Mycobacterium tuberculosis exhibits a significant reduction in its ability to adhere to and invade A549 pulmonary epithelial cells, with decreases of 69.39% (*p* = 0.047) and 56.20% (*p* = 0.033), respectively.	[60]
		N.A.	Adhesion to and invasion of macrophages are reduced by 42.16% (*p* = 0.107) and 69.02% (*p* = 0.052), respectively, in the pili-deficient Δmtp mutant compared to the wild-type.	[61]
		Laminin	MTP binds to the extracellular matrix protein laminin and contributes to biofilm formation. Isogenic mtp mutants lose the ability to produce MTP in vitro and demonstrate decreased laminin-binding capabilities.	[62,63]
12	19-kDa antigen	DC-SIGN	N.A.	[64]
		Mannose receptor	The 19-kDa antigen (Rv3763) binds to THP-1 macrophages through the macrophage mannose receptor (MR), promoting mycobacterial uptake.	[65]
13	Malate synthase (GlcB)	Laminin, Fn	Antibodies targeting the C-terminal laminin/fibronectin-binding domain inhibit the binding of *M. tuberculosis* to laminin and fibronectin, significantly reducing its adherence to A549 lung epithelial cells.	[66]
14	Glyceraldehyde-3-phosphate dehydrogenase (GAPDH)	Plg, Plm	Enhances bacterial binding to and translocation across lung epithelial cell barriers.	[67]
15	Alanine and proline-rich protein (Apa)	DC-SIGN	N.A.	[64]
		Fibronectin	Peptides 177-201 and 269-292 of Apa-A specifically bind fibronectin, with peptide 269-292 additionally inhibiting full-length protein interactions.	[68]
		human pulmonary surfactant protein A (PSP-A)	Apa remains stably associated with the cell wall, providing a platform that facilitates the attachment of PSP-A, thereby enhancing bacterial adhesion to host surfaces.	[69]
16	PstS-1	Mannose receptor	N.A.	[70]
17	N-acetylmuramoyl-L-alanine amidase (*Rv3717*)	Fn, laminin	N.A.	[71]
18	Mycobacterial mammalian cell entry protein 1A (Mce1A)	N.A.	Mce1A protein is crucial for *Mycobacterium leprae* invasion into human microvascular endothelial cells (HMVECs), and antibodies targeting Mce1A may inhibit this process.	[72]
		N.A.	Mce1A enhances bacterial adhesion and intracellular survival, promoting infection in monocytes and 3D bovine enteroids.	[73,74]
19	GlnA1	Plg, Fn	GlnA1 binds to human Plg and facilitates its activation to Plm, while also interacting with the extracellular matrix protein fibronectin, potentially enhancing *M. tuberculosis* tissue invasion.	[57]
20	Mpt64	N.A.	N.A.	[57,75,76]
21	PE_PGRS33 (*Rv1818c*)	Toll-like receptor 2 (TLR2)	PE-PGRS33 binds to TLR2 on macrophages in a calcium-dependent manner, facilitating bacterial adhesion and entry.	[77,78,79]
22	PE_PGRS81 (Rv1759c)	Fn	PE-PGRS81 binds fibronectin via its C-terminal fragment and shows reactivity with sera from TB patients.	[80]

* Although ESAT-6 and CFP-10 are primarily characterized as secreted virulence factors, they are included here as non-canonical adhesins based on studies reporting their direct interaction with host cells and facilitation of bacterial entry or dissemination.

## Data Availability

Not applicable.

## Abbreviations

The following abbreviations are used in this manuscript:

**Glossary**	**Full Term**
AEC	Alveolar epithelial cells
AG	Arabinogalactan
AG–PG	Arabinogalactan–peptidoglycan
AM	Alveolar macrophages
Antigen 85	Ag85
ANN	Artificial neural network
Apa	Alanine and proline-rich protein
BLF	Bovine lactoferrin
CD1⁻ DCs	CD1-negative dendritic cells
CFP-10	Culture filtrate protein 10 kDa
CH	Conserved Hypothetica
COVID-19	Coronavirus disease
DC-SIGN	Dendritic cell-specific ICAM-3-grabbing non-integrin
ECM	Extracellular matrix
ESAT-6	Early secretory antigenic target 6 kDa
Fn	Fibronectin
GAPDH	Glyceraldehyde-3-phosphate dehydrogenase
GlcNAc	N-acetylglucosamine
GlcB	Malate synthase
HBHA	Heparin-binding hemagglutinin adhesin
HMVEC	Human microvascular endothelial cells
*H. pylori*	*Helicobacter pylori*
LAMs	Lipoarabinomannans
LMs	Lipomannans
MARCO	Macrophage receptor with collagenous structure
MAs	Mycolic acids
ManLAM	Mannose-capped lipoarabinomannan
*M. bovis* BCG	*Mycobacterium bovis Bacillus Calmette-Guérin*
Mce1A	Mycobacterial mammalian cell entry protein 1A
MDP	Muramyl dipeptide
Mincle	Macrophage-inducible C-type lectin
MR	Macrophage mannose receptor
MTP	*Mycobacterium tuberculosis* curli pili
*M. tuberculosis*	*Mycobacterium tuberculosis*
TB	Tuberculosis
TDM	Trehalose 6,6′-dimycolate
TLR2	Toll-like receptor 2
TLRs	Toll-like receptors
TMM	Trehalose monomycolate
UN	United Nation
UPEC	Uropathogenic *Escherichia coli*
P_ad_	Protein being an adhesin
PAMPs	Pathogen-associated molecular patterns
PDIM	Phenolic glycolipid
PG	Peptidoglycan
PIMs	Phosphatidylinositol mannosides
Plg	Plasminogen
PRRs	Pattern recognition receptors
PSP-A	Human pulmonary surfactant protein A
SPAAN	Software Program for Prediction of Adhesins and Adhesin-like Proteins using Neural Networks
WHO	World Health Organization
